# Sympathetic innervation of inguinal white adipose tissue in the mouse

**DOI:** 10.1002/cne.25031

**Published:** 2020-09-28

**Authors:** Clara Huesing, Emily Qualls‐Creekmore, Nathan Lee, Marie François, Hayden Torres, Rui Zhang, David H. Burk, Sangho Yu, Christopher D. Morrison, Hans‐Rudolf Berthoud, Winfried Neuhuber, Heike Münzberg

**Affiliations:** ^1^ Neurobiology of Nutrition and Metabolism Department Pennington Biomedical Research Center, Louisiana State University System Baton Rouge Louisiana USA; ^2^ Institute for Anatomy and Cell Biology, Friedrich‐Alexander University Erlangen Germany

**Keywords:** iDISCO, imaging, pseudorabies virus, sympathetic chain ganglia, tissue clearing, transsynaptic retrograde tracing

## Abstract

Adipose tissue plays an important role in metabolic homeostasis and its prominent role as endocrine organ is now well recognized. Adipose tissue is controlled via the sympathetic nervous system (SNS). New viral, molecular‐genetic tools will soon allow a more detailed study of adipose tissue innervation in metabolic function, yet, the precise anatomical extent of preganglionic and postganglionic inputs to the inguinal white adipose tissue (iWAT) is limited. Furthermore, several viral, molecular‐genetic tools will require the use of cre/loxP mouse models, while the available studies on sympathetic iWAT innervation were established in larger species. In this study, we generated a detailed map for the sympathetic innervation of iWAT in male and female mice. We adapted iDISCO tissue clearing to process large, whole‐body specimens for an unprecedented view of the natural abdominal SNS. Combined with pseudorabies virus retrograde tracing from the iWAT, we defined the preganglionic and postganglionic sympathetic input to iWAT. We used fluorescence‐guided anatomical dissections of sympathetic nerves in reporter mice to further clarify that postganglionic axons connect to iWAT via lateral cutaneous rami (dorsolumbar iWAT portion) and the lumbar plexus (inguinal iWAT portion). Importantly, these rami carry axons that branch to iWAT, as well as axons that travel further to innervate the skin and vasculature, and their functional impact will require consideration in denervation studies. Our study may serve as a comprehensive map for future experiments that employ virally driven neuromodulation techniques to predict anatomy‐based viral labeling.

## Graphical abstract

- Preganglionic and postganglionic sympathetic inguinal white adipose tissue (iWAT) inputs are anatomically distinct from interscapular brown adipose tissue.
- Dorsolumbar and inguinal iWAT portions are innervated by anatomically distinct sympathetic nerves.
- Sympathetic postganglionic iWAT innervation reaches its target organ via lateral cutaneous rami of intercostal nerves T11–L1 and anterior cutaneous femoral nerve (as part of the lumbar plexus, L1)
- White adipocytes are sparsely innervated (sparse varicosities), while brown adipocytes (beige islands) show dense innervation (dense varicosities) in iWAT.

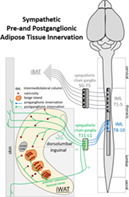

## 1 INTRODUCTION

Historically, white adipose tissue (WAT) was largely considered a storage depot for excess energy (Steiner & Cahill, 1964). However, given the increasing number of obese individuals plagued with metabolic syndrome, and the associated risk for life‐threatening comorbidities like Type 2 diabetes, cancer, and cardiovascular disease (Eyre et al., 2004), intense research over the last decades has highlighted a more detailed picture of the sophisticated structures within adipose tissue. Mature adipocytes co‐exist with connective tissue, stromavascular cells, immune cells as well as nerve fibers and terminals; all of these are involved in endocrine crosstalk within the tissue and across other organs (Kershaw & Flier, 2004).

Just as excess adipose tissue poses clear health risks, the lack of adipose tissue, lipodystrophy, also leads to severe metabolic dysfunction, like liver steatosis and insulin resistance (Petersen et al., 2002). One of the main functions of adipocytes is to switch between energy storage in times of excess through lipogenesis and fat storage and serving as an energy source via lipolysis to provide energy during periods of low food intake (e.g., sleep, starvation). Since the sympathetic nervous system (SNS) plays a critical role for the induction of lipolysis in adipocytes (Bartness, Shrestha, Vaughan, Schwartz, & Song, 2010; Leboeuf, Flinn, & Cahill, 1959; Weiss & Maickel, 1968), it is a good candidate for obesity interventions.

Previous studies have used diverse tracing strategies in various animal models to identify the origin of sympathetic neurons that innervate the adipose tissue. In Siberian hamsters, the retrograde tracer, fluorogold was injected into inguinal WAT (iWAT) and identified labeled cell bodies in the abdominal sympathetic chain ganglion T13 (Youngstrom & Bartness, 1995). Later studies used the transsynaptic retrograde tracer, pseudorabies virus (PRV), in rats and showed labeling in the abdominal sympathetic chain ganglia (SChG) T13 and L1 (Wiedmann, Stefanidis, & Oldfield, 2017). Conversely, a retrograde tracing study with Cholera toxin b conducted in mice suggested that iWAT receives sympathetic inputs from the celiac ganglion (CG; Jiang, Ding, Cao, Wang, & Zeng, 2017).

Given these inconsistent findings and the increased interest to target the peripheral nervous system with novel molecular genetic tools (Ch'ng & Enquist, 2005; Francois et al., 2019; Jiang et al., 2017; Pereira et al., 2017; Wiedmann et al., 2017), the mouse will likely be the model of choice to target sympathetic innervation of iWAT. However, the mouse has not been a preferred model for dissection of the peripheral nervous system due to its small size. Published literature regarding the organization of the SNS in rodents is typically available from rats (Baron, Janig, & Kollmann, 1988). Yet, in order to apply novel molecular‐genetic and viral tools in the peripheral nervous system, the mouse is the model of choice due to the abundant availability of cre‐driver and floxed mouse lines.

In this study, we aimed to develop a method that allows to recapitulate the anatomical context of sympathetic innervation of iWAT in male and female mice. In a recent study, we adapted the tissue clearance and immunolabeling methodology, iDISCO (immunolabeling‐enabled three‐dimensional imaging of solvent‐cleared organs, Renier et al., 2014), to clear the entire spinal cord (SC) and associated peripheral ganglia in the thorax and abdomen (Francois et al., 2019; Münzberg et al., 2019). This technique allowed for high‐resolution imaging of the SNS within the abdomen, all the while leaving the SChG largely intact and undisturbed. In this study, we combined whole body iDISCO with the retrograde transsynaptic tracer PRV to identify preganglionic and postganglionic sympathetic inputs to iWAT. We further include anatomical dissection techniques in reporter mice to provide a detailed anatomical map for the sympathetic innervation of iWAT.

Our PRV study clarifies the origin of postganglionic iWAT innervation from SChG T12–L1 and preganglionic inputs from the SC levels T7–T10 in the mouse. We show that the lateral cutaneous rami continue from iWAT to the skin, while fine nerves containing sympathetic axons branch off to the dorsolumbar portion of iWAT. Another major innervation is provided by the anterior and lateral femoral cutaneous nerves to the inguinal portion of iWAT.

## 2 MATERIALS AND METHODS

Data associated with this study and François et al., 2019 (data set Münzberg et al., 2019) were collected as part of the Stimulating Peripheral Activity to Relieve Conditions (SPARC) project and are or will be made available through the SPARC Data Portal (RRID: SCR_017041) under a CC‐BY 4.0 license.

### 2.1 Animals

TH‐IRES‐Cre mice (EM: 00254; B6.129X1‐Th^tm1(Cre)Te^/Kieg; European Mouse Mutant Archive; breeding pairs were obtained from Dr Luis de Lecea, Stanford University) were crossed with Rosa‐EGFP^*fl/fl*^ reporter mice (B6;129S4‐Gt(ROSA)26Sor<tm9(EGFP/Rpl10a)Amc>/J, Stock# 024750, Jackson Laboratory, Bar Harbor, ME) or Rosa‐Tomato^*fl/fl*^ mice (stock #: 007914; B6.Cg‐Gt(ROSA)26Sor^tm14(CAG‐tdTomato)Hze^/J, Jackson Laboratories, Bar Harbor, ME) to generate tyrosine hydroxylase–enhanced green fluorescent protein (TH:EGFP) or TH:Tomato reporter mice. Animal genotypes were confirmed by polymerase chain reaction (PCR) from tail biopsies DNA (TH‐IRES‐Cre: Cre reverse 5′‐GAT‐ACC‐TGG‐CCT‐GGT‐CTG‐3′; wild‐type/Cre forward 5′‐CAC‐CCT‐GAC‐CCA‐AGC‐ACT‐3′; wild‐type reverse 5′‐CTT‐TCC‐TTC‐CTT‐TAT‐TGA‐GAT‐3′; Rosa‐EGFP^*fl/fl*^ mice: wild‐type forward 5′‐AAG GGA GCT GCA GTG GAG TA‐3′; wild‐type reverse 5′‐CCG AAA ATC TGT GGG AAG TC‐3′; mutant forward 5′‐ATT GCA TCG CAT TGT CTG AG‐3′; mutant reverse 5′‐CCG AAA ATC TGT GGG AAG TC‐3′; Rosa‐Tomato^*fl/fl*^ mice: wild‐type forward 5′‐AAG GGA GCT GCA GTG GAG TA‐3′; wild‐type reverse 5′‐CCG AAA ATC TGT GGG AAG TC‐3′; mutant forward 5′‐CTG TTC CTG TAC GGC ATG G‐3′; mutant reverse 5′‐GGC ATT AAA GCA GCG TAT CC‐3′). Both male and female mice were used in all experiments and all animals were group‐housed at a 12:12‐hr light/dark cycle with ad lib access to food and water unless stated otherwise. The Institutional Animal Care and Use Committee approved all animal experiments.

### 2.2 Fluorescence‐guided dissection of tissue

We initially performed anatomical dissections in order to clarify the overall organization of the lower thoracic and upper abdominal SNS as well as iWAT innervation. We used TH:GFP (*n* = 1) and TH:tomato reporter (*n* = 6, 3 males, 3 females; ranging from 6 to 20 weeks of age) lines for dissections under a fluorescent stereomicroscope (Nikon, SMZ25, Melville, NY). Mice were euthanized with an overdose of CO_2_ and blood was removed by cardiac perfusion with saline. Thoracic and abdominal organs were removed and images of the prevertebral and paravertebral structures were taken for documentation. In some cases, we carefully removed the adipose tissue that surrounds all sympathetic ganglia (“ganglia fat”) to enhance visibility and imaging of abdominal ganglia. We further identified and documented major innervating nerves and their origin by dissection under the fluorescent stereomicroscope. In some cases we used TH:tomato mice to enhance visibility of fine nerves.

### 2.3 PRV infection of iWAT

Mice (ranging from 6 to 15 weeks old) were anesthetized with isoflurane/oxygen. A lower back incision was extended laterally on the right hind leg to reveal an adequate amount of iWAT sufficient for injection. Ten individual injections (each 100 nl, *n* = 2 or 200 nl, *n* = 11) were distributed across the right iWAT depot with green fluorescent protein expressing PRV (PRV‐GFP, viral titer, 1 × 10^9^ viral molecules/ml, Lot #2007, kindly provided by the National Center for Experimental Neuroanatomy with Neurotropic Viruses; Pittsburgh, PA). Virus was injected with a beveled Hamilton® syringe (Model 7002 KH SYR, Reno, NV). Upon each injection, the syringe was held in place for 30–40 s to prevent backflow. Injection sites were dried with a cotton swab to mitigate leakage to surrounding tissue and circulation. Mice were single‐housed postviral infection for 96 hr. Note that our goal was to use minimal infection times, and we tested 10 × 100 nl injection with different incubation times of 72 hr (*n* = 9) and 96 hr (*n* = 5), with no infection at 72 hr and only 2 successfully infected animals at 96 hr. Doubling the viral injection volume at 96‐hr incubation time increased the rate of successful infections (as observed under the dissecting microscope, 11/13). Control animals involved dripping the same total volume of PRV‐GFP (1 μl, *n* = 1 or 2 μl, *n* = 4, 96‐hr incubation) onto the surface of the iWAT depot.

Perfusion and immunohistochemistry were performed as previously described (Francois et al., 2019). Mice were deeply anesthetized with an overdose of isoflurane, followed by a transcardiac syringe perfusion with ice‐cold physiological saline, then 10% neutral buffered formalin (Fisher, Passaic, NJ). Thoracic and abdominal organs were removed, which allowed for verification of successful infection when viewed with a fluorescent stereomicroscope (Nikon, SMZ25, Melville, NY). Only mice with visible SChG infection were included in further tissue dissection and analysis of SChG (*n* = 13, 6 females, 7 males), intermediolateral nucleus of the SC (IML) (*n* = 11, 4 females, 7 males), dorsal root ganglia (DRG) (*n* = 13, 6 females, 7 males), and CG (*n* = 8, 5 females, 3 males). The iWAT was removed for individual processing and excessive muscle mass was dissected from the SC. A laminectomy was performed in order to image sympathetic preganglionic neurons. The spine and SC were cut in half (roughly at the level of ribs 7–9) to accommodate the imaging capacity of both the light sheet and confocal microscope. Then, the tissue was postfixed overnight in formalin, and stored in phospate buffered saline (PBS)–azide (0.02% Na–azide in PBS) 4°C until iDISCO processing. A detailed experimental protocol is available through Protocols.io (https://www.protocols.io/private/AC95D1B52DAFBB09536341A0B6668D33).

### 2.4 Immunohistochemistry and tissue clearing

Immunohistochemical staining was performed following the iDISCO method from Renier et al. (https://idisco.info/idisco-protocol/update-history/) with modifications. Tissues were dehydrated in a series of methanol (MeOH)/H_2_O mixtures (20, 40, 60, 80, and 100%), then incubated overnight in a 66% dichloromethane (DCM) and 33% MeOH solution on a rocker (Southwest Science, model SBT30, Trenton, NJ). Tissues were treated with 5% H_2_O_2_ in MeOH overnight at 4°C. Following overnight incubation, samples were rehydrated in a series of 1‐hr MeOH/H_2_O washes, then washed in PTx.2 solution (0.2% TritonX‐100 in PBS). Samples were incubated in permeabilization solution (400 ml PTx.2, 11.5 g glycine, 100 ml dimethylsylfoxid (DMSO)), followed by incubation in blocking solution (42 ml PTx.2, 5 ml DMSO, 3 ml normal donkey serum (NDS)) at 37°C for 2 days in a shaking incubator (Corning LSE Product #6790, Corning, NY). Then, tissues were incubated with primary antibodies (chicken anti‐GFP [1:400], Abcam, Cambridge, MA; rabbit anti‐TH [1:400], Millipore, Burlington, MA; CD31/PECAM‐1 [1:300], Novus Biologicals, Littleton, CO) in primary incubation solution (92 ml PTwH (1 ml of 10 mg/ml heparin stock solution, 2ml Tween‐20, *quantum satis* to 1 liter with PBS), 5 ml DMSO, 3 ml NDS) on a shaking incubator at 37°C for at least 7 days. Following primary staining, tissues underwent five, 60‐min washes in PTwH then subjected to secondary staining (AlexaFluor 647 donkey anti‐chicken [1:300], AlexaFluor 555 donkey anti‐rabbit [1:300], Jackson ImmunoResearch, West Grove, PA, AlexaFluor 488 donkey anti‐goat [1:300], ThermoFisher Scientific, Waltham, MA) in secondary incubation solution (PTwH with 3% NDS) for at least 7 days at 37°C on a shaking incubator. Specific labeling of primary antibodies was confirmed in this experiment based on initial visual stereomicroscopic inspection of PRV^GFP^ endogenous fluorescence in SChG of unstained specimen, for anti‐TH by restricted labeling of SChG and their axons, and for anti‐CD31 by restricted labeling of endothelial structures. Once labeling was confirmed, samples underwent the same PTwH wash step, then an additional round of MeOH dehydration (both washes described above). Tissues were incubated in 100% MeOH overnight and then submerged in 66% DCM/33% MeOH solution for 3 hr. Once completed, the samples underwent two, 100% DCM washes, and were then placed in dibenzyl ether (DBE), an organic solvent, for at least 3 hr at room temperature for clearing. A detailed experimental protocol is available through Protocols.io (https://www.protocols.io/private/B7569F5C0050B3198C8BBC721EC210BC). Additional antibody information can be found in Table 1.

**TABLE 1:** List of used resources

Reagent type, species	Source, catalog number	Product name	Host	Target	Immunogen	Amino acid sequence	Monoclonal or polyclonal	Specificity	Positive control	Concentration	RRID
Antibody	Abcam, ab13970	Anti‐GFP	Chicken		Recombinant full length protein corresponding to GFP from *Aequorea Victoria*	UniProt ID: P42212	Polyclonal	IgY	Tissues from transgenic animals expressing GFP and cultured cells transfected with GFP (Zhang et al., 2011)	(1:400)	AB_300798
Antibody	Millipore, AB152	Anti‐tyrosine hydroxylase	Rabbit		Denatured tyrosine hydroxylase from rat pheochromocytoma (denatured by sodium dodecyl sulfate)	UniProt ID: P04177	Polyclonal	AB152 selectively labels a single band at approximately 62 kDa (reduced) corresponding to tyrosine hydroxylase	Brain (corpus striatum, sympathetic nerve terminals) and adrenal glands (see also Tabuchi et al., 2007)	(1:500)	AB_390204
Antibody	Novus Biologicals, AF3628	CD31/ PECAM‐1	Goat		Mouse myeloma cell line NS0‐derived recombinant mouse	UniProt ID: Q08481	Polyclonal	IgG	CD31/PECAM‐1 was detected in immersion fixed bEnd.3 mouse endothelioma cell line using goat anti‐mouse/rat CD31/PECAM‐1 antigen affinity‐purified polyclonal antibody (catalog # AF3628) at 10 μg/ml for 3 hr at room temperature. Cells were stained using the NorthernLightsTM 493‐conjugated anti‐goat IgG secondary antibody (green; catalog # NL003) and counterstained with DAPI (blue). Specific staining was localized to cell membrane.	(1:300)	B_2161028
Antibody	Thermo Fisher Scientific, A‐11055	Anti‐goat IgG (H + L) cross‐adsorbed secondary antibody Alexa Fluor 488	Donkey	Goat	Gamma Immunoglobins, heavy and light chains		Polyclonal	IgG		(1:300)	AB_2534102
Antibody	Thermo fisher scientific, A‐31572	Anti‐rabbit IgG (H + L) highly cross‐adsorbed secondary antibody Alexa Fluor 555	Donkey	Rabbit	Gamma Immunoglobins, heavy and light chains		Polyclonal	IgG		(1:300)	AB_162543
Antibody	Jackson ImmunoResearch Labs, 703–606‐155	Alexa Fluor 647‐AffiniPure F(ab’)2 fragment anti‐chicken IgY (IgG) (H + L)	Donkey	Chicken	Gamma Immunoglobins, heavy and light chains		Polyclonal	IgY		(1:300)	AB_2340380
Genetic reagent (*M. musculus*)	European Mouse Mutant Archive, EMMA ID: 00254	B6.129X1‐Thtm1(cre)Te/Kieg									IMSR_EM: 00254
Genetic reagent (M. musculus)	Jackson Laboratories, stock no: 007914	B6.Cg‐Gt(ROSA)26 Sortm14(CAGtdTomato)Hze/J									IMSR_JAX: 007914
Genetic reagent (M. musculus)	Jackson Laboratories, Stock no: 024750	B6;129S4‐Gt(ROSA)26 Sortm9(EGFP/Rpl10a)Amc/J									IMSR_JAX: 024750
Genetic reagent (Pseudorabies virus)	Center for Neuroanatomy with Neurotropic Viruses	Pseudorabies virus (PRV)‐152 (Bartha) containing the CMV‐EGFP reporter gene cassette inserted into the gG locus of the viral genome									
Data processing software	Oxford Instruments	Imaris									SCR_007370

### 2.5 Microscopy and image processing

A Leica SP5, SP8 X (Leica Biosystems Inc., Buffalo Grove, IL), or Ultramicroscope II (LaVision BioTec, Bielefeld, Germany) were used to image all relevant structures. To allow for confocal imaging of DBE‐cleared tissues, a chamber was manufactured in‐house. Three‐dimensional (3D) overview images were generated of the spine and SC in order to reveal PRV labeling in preganglionic sympathetic neurons (in the IML and medially) and DRG. The spine was repositioned with the ventral side up to allow for optimal imaging of thoracic and lumbar SChG, as well was the CG. Lightsheet imaging was performed with a zoom factor of 0.63× or 1.6× (specified in figure captions). Images of TH and PRV labeling were collected for all samples, while CD31 staining was not performed for all samples. Images from the far‐red fluorescent channel were pseudocolored as green. Additionally, higher magnification (10×/0.40 NA) confocal images captured of PRV‐positive SChG, their corresponding DRG, and PRV‐positive preganglionic cell bodies. A detailed experimental protocol is available through Protocols.io (https://www.protocols.io/edit/light-sheet-microscopy-wz3ff8n).

## 3 RESULTS

### 3.1 TH‐IRES‐cre/tomato mice reveal the organization of prevertebral and paravertebral ganglia in the thoracic and abdominal cavities

**FIGURE 1:**
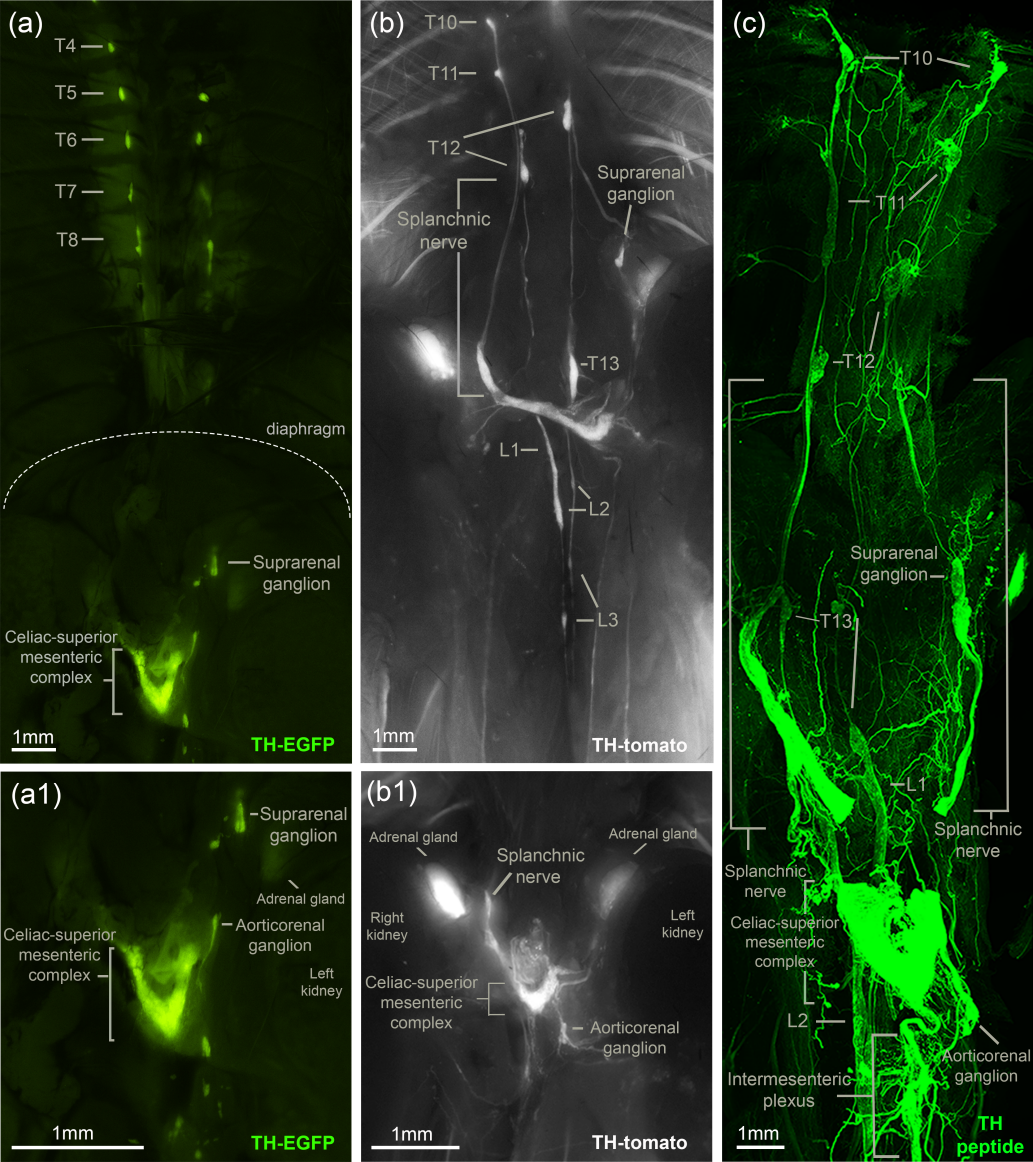
Ventral views of sympathetic chain and prevertebral ganglia as seen under the fluorescence dissecting microscope (a–c) and in the light sheet microscope after iDISCO processing (d). (a,a1) TH‐EGFP reporter mouse (male, 8 weeks old, 0.5× objective) with ventral view of the SChG (T4–T8) and abdominal prevertebral sympathetic ganglia (celiac superior mesenteric complex) at low magnification. Note that the lower SChG caudal to T8 are obscured by overlying fat tissue. The celiac superior mesenteric complex depicts the fused celiac and superior mesenteric ganglia as well as the left suprarenal, the left aorticorenal ganglia (a1). (b,b1) TH‐tomato mouse (male, 12 weeks old, 0.5× objective) with ventral views of lower thoracic SChG (T10–T13) and upper lumbar SChG L1–L3. The lumbar SChG can be seen after moving the overlying prevertebral ganglia to the side. In (b1) the celiac superior‐mesenteric complex is seen undisturbed, showing several details, for example, splanchnic nerves and aorticorenal ganglion. Note several non‐specifically labeled structures such as ribs and muscle fibers. (c) Light sheet microscope extended focus view of cleared tissue block (iDISCO, 1.6× zoom factor) with tyrosine hydroxylase (TH‐peptide) immunohistochemistry from a 12‐week‐old, male mouse, showing the lower thoracic and upper lumbar chain ganglia, together with the overlaying celiac‐superior mesenteric complex and connecting nerves. Note the detailed view of lower sympathetic chain ganglia, greater splanchnic nerves entering suprarenal and celiac‐superior mesenteric ganglionic complex. Also note the left aorticorenal ganglion and intermesenteric plexus. A long interganglionic strand links sympathetic chain ganglia T12 and T13 through the diaphragm. EGFP, enhanced green fluorescent protein; SChG, sympathetic chain ganglia; TH, tyrosine hydroxylase [Color figure can be viewed at wileyonlinelibrary.com]

We aimed to visualize and document the overall organization of the thoracic and abdominal SNS in adult mice and utilized cre‐driver mice for the sympathetic marker TH, to express the reporter genes EGFP‐L10a (TH:EGFP mice) or tdTomato (TH:tomato mice). The ribosomal protein L10a targets EGFP expression to the cell body, so that TH:EGFP mice show strong labeling of cell body containing paravertebral ganglia (SChG) and prevertebral ganglia (celiac, suprarenal, aorticorenal, and superior mesenteric ganglia); but no labeling in nerve fibers (Figure 1a,a1). In contrast, the cytoplasmatic tdTomato expression labels cell bodies and nerve fibers, revealing the connectivity of sympathetic chain and celiac complex in TH:tomato mice (Figure 1b,b1). In the mouse, the splanchnic nerve consistently branched off at the level of the sympathetic chain ganglion T12 and the elongated interganglionic strands between T12 and T13 (piercing through the diaphragm) served as a prominent landmark to identify upper abdominal chain ganglia T13, and lumbar ganglia L1–L3 (Figure 1b). Abdominal chain ganglia required careful dissection to allow visualization for imaging (Figure 1b) as overlaying adipose tissue often obscured or blocked the fluorescent signal (Figure 1a, ganglia caudal to T8 not visible). To fully visualize the abdominal chain ganglia, the celiac‐superior mesenteric complex was moved rostral in Figure 1b, while the undisturbed view of the celiac complex is shown in Figure 1b1 with further details of the aorticorenal ganglia and splanchnic nerves.

### 3.2 Large‐volume tissue clearance reveals 3D anatomy of SNS

Tissue clearing is a well‐established technique used to make an otherwise deep, opaque sample optically transparent to improve anatomical analysis. In 2014, Renier et al. modified the existing 3DISCO clearing protocol to allow the use of immunofluorescence labeling in large solvent‐cleared tissues (Renier et al., 2014). We modified the iDISCO method to perform whole body histological staining for TH‐peptide and tissue clearing in adult mice, allowing for remarkable visualization of SChG and their physical connections, as well as fine innervation structures of the celiac‐superior mesenteric plexus (Figure 1c). We also note that this provides unparalleled anatomical detail and context, which garners precise and comprehensive reporting. Furthermore, TH‐peptide immunostaining is a more reliable marker of the SNS compared to direct TH‐cre‐driven reporters. During early development, tyrosine hydroxylase is transiently expressed in cholinergic parasympathetic neurons (Howard, 2005), meaning that parasympathetic neurons may also be labeled in TH‐reporter mice with TH‐cre induced reporter expression throughout development. Figure 1c clearly demonstrates TH‐peptide staining in iDISCO‐cleared tissue verifying the abdominal sympathetic organization.

### 3.3 iWAT is innervated by postganglionic neurons located in SChG T12, T13, and L1

**FIGURE 2:**
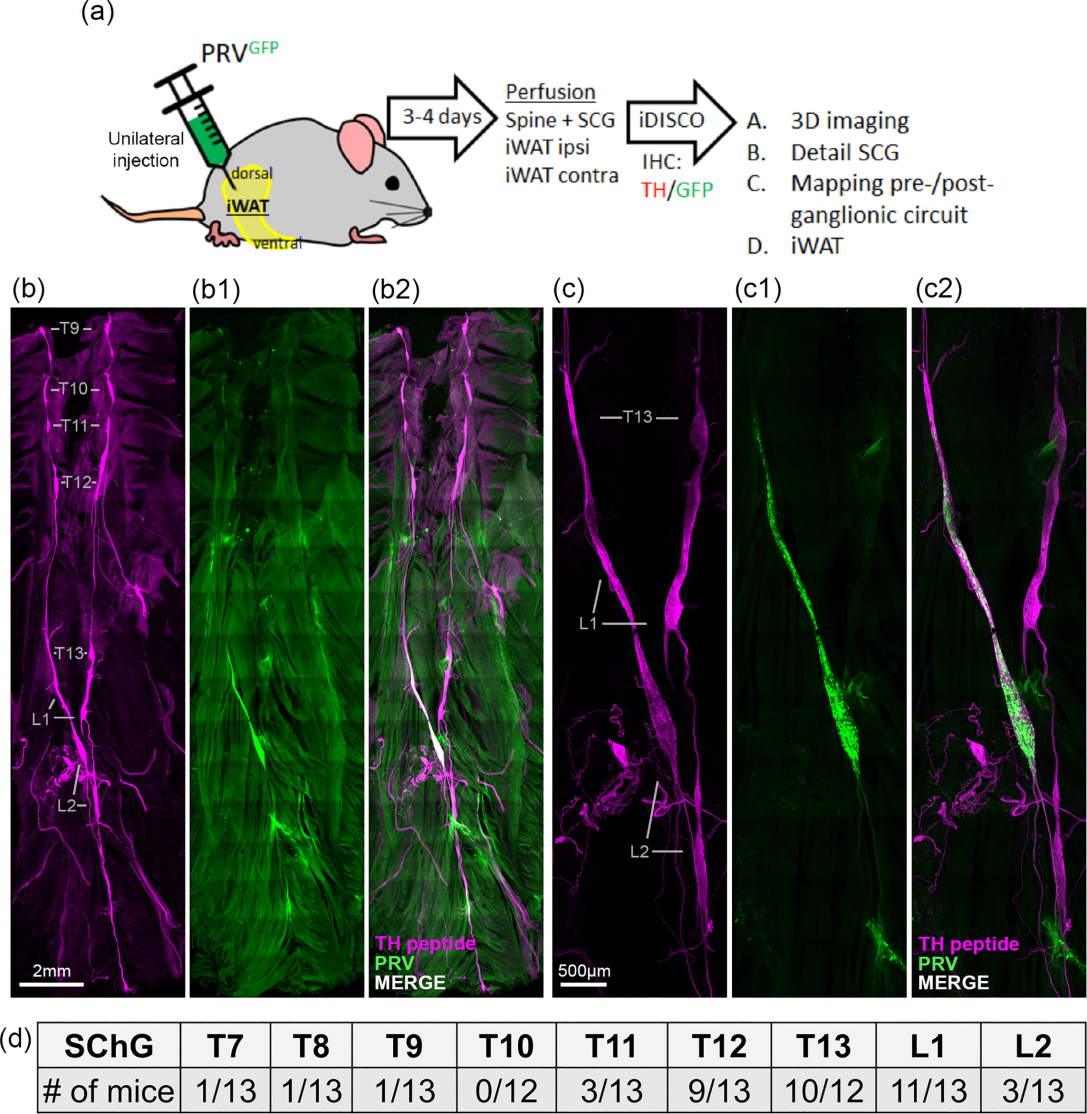
Sympathetic postganglionic innervation of iWAT. (a) Mice were injected with the retrograde, transsynaptic tracer PRV^GFP^ into the right iWAT pad. Perfusion and tissue harvest was performed 3–4 days postviral injection. Whole body iDISCO for TH peptide (b and c) and PRV ((b1) and (c1) imaged under a confocal microscope (10× objective) of a 12‐week‐old female mouse). In the merged image (b2, c2) retrogradely labeled postganglionic sympathetic neurons can be seen in the right chain ganglia, T13 and L1. (d) Summary of ganglionic labeling (positive cases/total number of mice) showing most consistent labeling in T12 to L1. iWAT, inguinal white adipose tissue; PRV, pseudorabies virus; TH, tyrosine hydroxylase

We aimed to clarify the origin of sympathetic innervation of iWAT using iWAT‐derived labeling with fluorescent PRV (PRV^GFP^) and iDISCO TH‐peptide and GFP double staining. These methods provide a comprehensive analysis of overall sympathetic structures and their according labeling with PRV. We chose a minimal PRV infection time that was long enough to allow consistent labeling of SChG (4 days, Figure 2a), but was too short to label supraspinal sympathetic premotor neurons (e.g. raphe pallidus). Under these conditions, labeling was restricted to postganglionic and preganglionic neurons and spinal interneurons.

Light sheet and confocal microscope imaging allowed for 3D visualization and analysis of the entire thorax and abdomen. Among the 13 analyzed animals (6 females, 7 males) with successful PRV labeling in SChG, we observed consistent labeling in ipsilateral SChG T12–L1 (Figure 2b–d), even though some variability was observed for individual cases showing labeling in T7–T9 (1/13), T11 (3/13), and L2 (3/13) (Figure 2d; Table 2). No tracer labeling was detected in contralateral ganglia.

**TABLE 2:** Individual results for all animals of pseudorabies virus (PRV)‐positive neurons found in sympathetic chain ganglia (a), dorsal root ganglia (b), celiac ganglia (c), Intermediolateral bundle (d)

PRV(GFP) labeling	(a) Sympathetic chain ganglia															
Animal	SG/T1	T2	T3	T4	T5	T6	T7	T8	T9	T10	T11	T12	T13	L1	L2	L3
WAT10 (F)												✓	✓	✓		
WAT12 (F)													✓	✓	✓	
WAT34 (F)												✓	✓	✓		
WAT35 (M)													No data	✓	✓	
WAT36 (M)												✓	✓	✓	✓	
WAT37 (M)													✓	✓		
WAT38 (M)														✓		
WAT40 (M)												✓	✓			
WAT56 (F)											✓	✓	✓	✓		
WAT57 (F)							✓	✓	✓	No data	✓	✓	✓			
WAT60 (M)											✓	✓	✓	✓		
WAT61 (M)												✓	✓	✓		
WAT62 (F)												✓		✓		
Male	0	0	0	0	0	0	0	0	0	0	1	4	5	5	2	0
Female	0	0	0	0	0	0	1	1	1	1	2	5	5	5	1	0
Total	0/13	0/13	0/13	0/13	0/13	0/13	1/13	1/13	1/13	0/12	3/13	9/13	10/12	11/13	3/13	0/13
Percent total (%)	0	0	0	0	0	0	8	8	8	0	23	69	83	85	15	0

PRV(GFP) labeling	(b) Dorsal root ganglia															
Animal	T1	T2	T3	T4	T5	T6	T7	T8	T9	T10	T11	T12	T13	L1	L2	L3
WAT10 (F)	No data	No data	No data	No data	No data	No data	No data	No data	No data	No data	✓	✓	✓			
WAT12 (F)	No data	No data	No data	No data	No data	No data	No data	No data	No data		✓	✓				
WAT34 (F)																
WAT35 (M)	No data	No data	No data	No data	No data	No data	No data	No data	No data							
WAT36 (M)																
WAT37 (M)																
WAT38 (M)																
WAT40 (M)																
WAT56 (F)										✓	✓					
WAT57 (F)				✓	✓	✓	✓	✓	✓	No data	✓	✓				
WAT60 (M)																
WAT61 (M)											✓					
WAT62 (F)									✓	✓	✓	✓	✓			
Male	0	0	0	0	0	0	0	0	0	0	1	0	0	0	0	0
Female	0	0	0	1	1	1	1	1	2	2	5	4	2	0	0	0
Total	0/10	0/10	0/10	1/10	1/10	1/10	1/10	1/10	2/10	2/11	6/13	4/13	2/13	0/13	0/13	0/13
Percent total (%)	0	0	0	10	10	10	10	10	20	18	46	31	15	0	0	0

PRV(GFP) labeling	(c) Celiac ganglia
Animal	
WAT10 (F)	
WAT12 (F)	No data
WAT34 (F)	No data
WAT35 (M)	No data
WAT36 (M)	No data
WAT37 (M)	No data
WAT38 (M)	No data
WAT40 (M)	No data
WAT56 (F)	
WAT57 (F)	
WAT60 (M)	✓
WAT61 (M)	
WAT62 (F)	✓
WAT66 (M)	
WAT67	No data
WAT68 (F)	
WAT69 (F)	No data
Male	1
Female	1
Total	2/8
Percent total (%)	25

PRV(GFP) labeling	(d) Intermediolateral nucleus of the spinal cord															
Animal	T1	T2	T3	T4	T5	T6	T7	T8	T9	T10	T11	T12	T13	L1	L2	L3
WAT10 (F)					✓	✓	✓	✓	✓	✓	✓					
WAT12 (F)					✓	✓	✓	✓	✓	✓	✓					
WAT34 (F)										✓						
WAT35 (M)							✓	✓	✓	✓	✓					
WAT36 (M)						✓	✓	✓	✓	✓	✓	✓				
WAT37 (M)						✓	✓	✓	✓	✓						
WAT38 (M)						✓	✓	✓	✓	✓						
WAT40 (M)					✓	✓	✓	✓	✓	✓						
WAT56 (F)	No data	No data	No data	No data	No data	No data	No data	No data	No data	No data	No data	No data	No data	No data	No data	No data
WAT57 (F)	No data	No data	No data	No data	No data	No data	No data	No data	No data	No data	No data	No data	No data	No data	No data	No data
WAT60 (M)								✓	✓	✓						
WAT61 (M)							✓	✓	✓							
WAT62 (F)					✓	✓	✓	✓	✓							
Male	0	0	0	0	1	4	6	7	7	6	2	1	0	0	0	0
Female	0	0	0	0	3	3	3	3	3	3	2	0	0	0	0	0
Total	0/13	0/13	0/13	0/13	4/11	7/11	9/11	10/11	10/11	9/11	4/11	1/11	0/13	0/13	0/13	0/13
Percent total (%)	0	0	0	0	36	64	82	91	91	82	36	9	0	0	0	0

The celiac complex was analyzed in 8 animals, and 6/8 animals (75%) showed no signs of PRV infection, even though one female and one male showed minimal to moderate PRV‐labeled cell bodies in the CG (data not shown). We concluded that the celiac‐superior mesenteric complex does not significantly contribute to iWAT innervation. In the rare cases with PRV labeling in the celiac‐superior mesenteric complex the injections may have unintentional punctured an indiscriminate area innervated by the celiac‐superior mesenteric complex or PRV may have diffused from subcutaneous depots through the (in rodents typically open) vaginal process of the inguinal canal (Berthoud, Fox, & Neuhuber, 2006).

**FIGURE 3:**
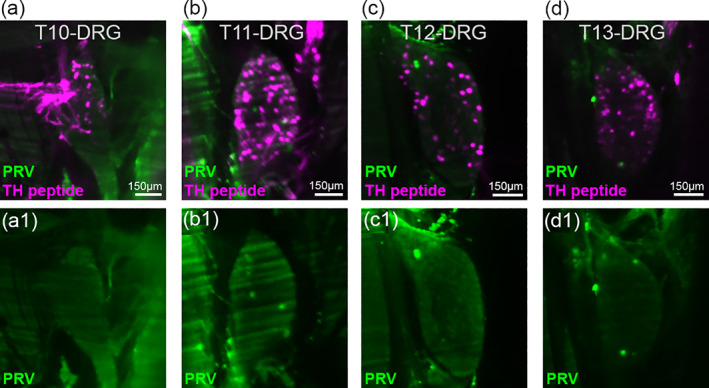
PRV labeling is occasionally found in dorsal root ganglia. Example of PRV labeling (green) in DRG at level T11 (a), T12 (b), T13 (c) and L1 (d). DRG neurons were easily identified by the existence of few, but consistent TH‐positive neurons (red). PRV‐positive DRG neurons were consistently not co‐localized with TH. Light sheet images, 0.63× zoom factor. DRG, dorsal root ganglia; PRV, pseudorabies virus; TH, tyrosine hydroxylase [Color figure can be viewed at wileyonlinelibrary.com]

Next, we looked for evidence of PRV labeling in DRG sensory neurons. In a similar study of interscapular brown adipose tissue‐derived PRV labeling, we identified a low, but consistent PRV labeling in the DRG (Francois et al., 2019). In contrast to interscapular brown adipose tissue (iBAT)‐related labeling (Francois et al., 2019; Münzberg et al., 2019), DRG ganglia showed only occasional PRV labeling with typically <5 neurons per ganglion and most often observed in DRG T11 and T12 in 46 and 30%, respectively, of all analyzed animals (Figure 3, Table 2). We note that iWAT‐derived PRV was not co‐localized with TH‐positive DRG neurons which lack authentic catecholaminergic functions (Kummer, Gibbins, Stefan, & Kapoor, 1990). Our finding is in line with another report indicating that TH‐positive DRG neurons, which often contained also calcitonin‐gene‐related peptide (CGRP), may rather innervate the colorectum and urinary bladder although the majority of CGRP‐positive axons in these organs apparently lacked TH immunoreactivity (Brumovsky, La, McCarthy, Hokfelt, & Gebhart, 2012).

**FIGURE 4:**
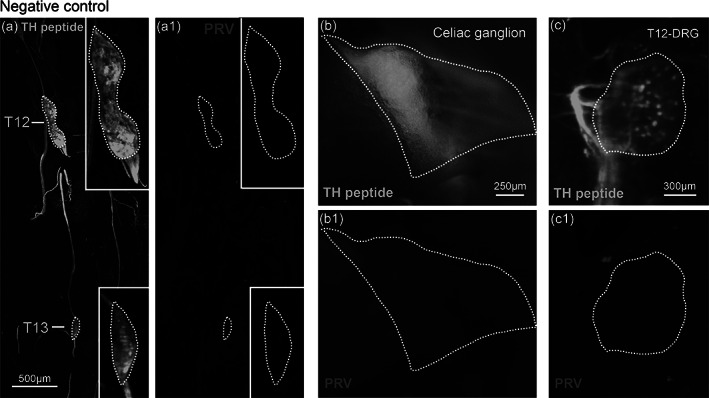
PRV injections into iWAT exclusively labels the sympathetic chain ganglia and IML. (a–c) Negative control “drip‐on” tests were conducted in five mice. PRV administered onto the iWAT via this method provided no evidence of PRV labeling in the sympathetic chain ganglia ((a,a1) light sheet microscope, 0.63× zoom factor), celiac ganglion ((b,b1) dissecting microscope, 0.5× objective), or iWAT ((c,c1) light sheet microscope, 0.63× zoom factor). IML, intermediolateral nucleus of the spinal cord; iWAT, inguinal white adipose tissue; PRV, pseudorabies virus

We further ensured that “off‐target” labeling was not due to an overall viral leakage, and dripped the same volume and titer of PRV onto iWAT tissue. In all five negative control animals, virus drip‐on was insufficient to infect SChG (Figure 4a,a1), celiac‐superior mesenteric complex (Figure 3b,b1), and DRG (Figure 3c,c1) with PRV.

### 3.4 Preganglionic sympathetic neurons innervating iWAT are located in the IML of the SC spanning from T7 to T10

**FIGURE 5:**
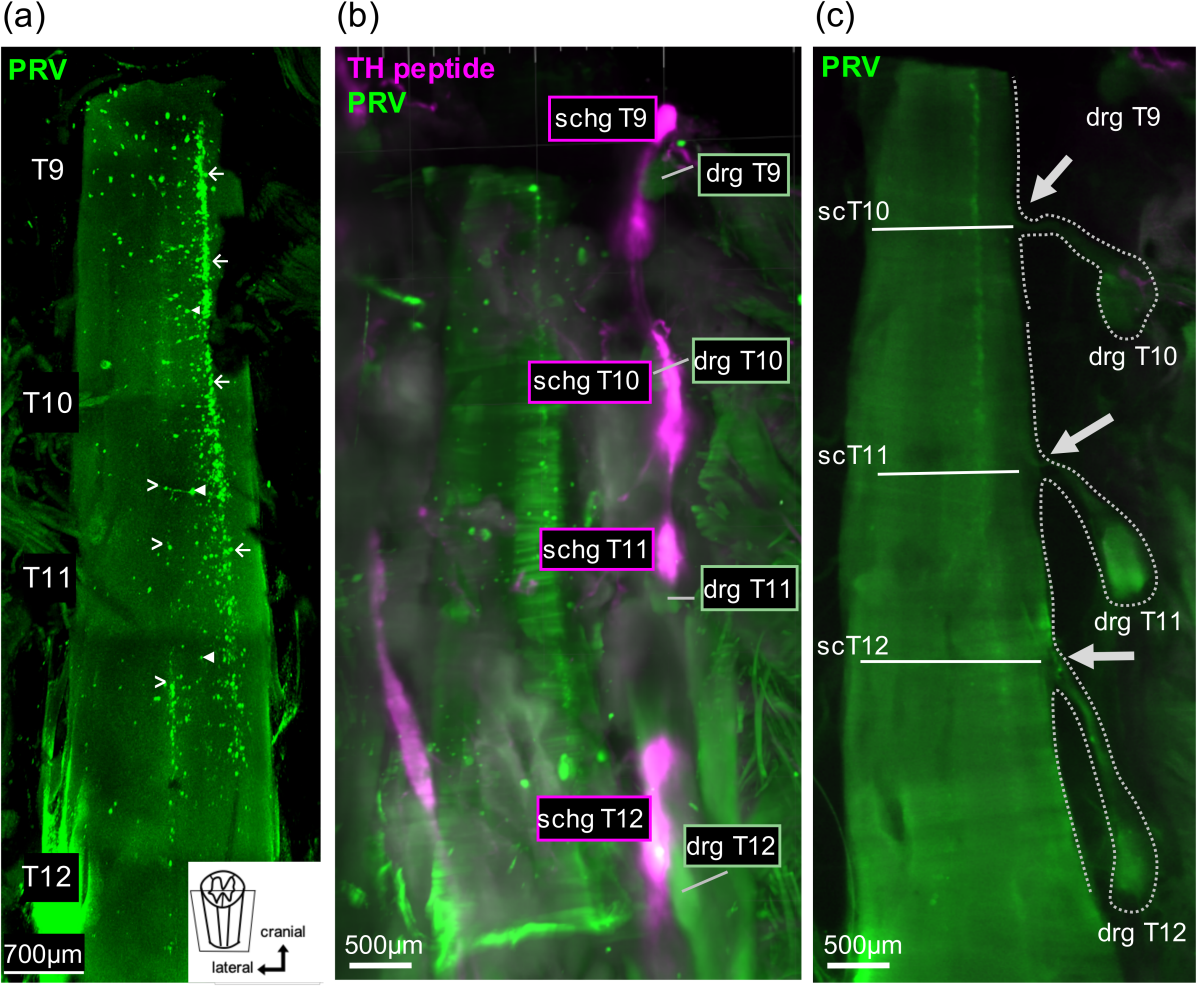
Preganglionic sympathetic neurons related to inguinal white adipose tissue (iWAT) in the spinal cord in iDISCO preparation. (a) Most preganglionic neurons‐labeled transsynaptically with pseudorabies virus (PRV) from iWAT are located in the intermediolateral nucleus (IML, white arrows), while a significant number is detected in the intercalated (IC, filled arrow heads) and central autonomic nuclei (CA, open arrowheads); confocal microscope, 10× objective. (b) In a 3D stack with overlaying tyrosine hydroxylase (TH) immunofluorescence, the levels of sympathetic chain ganglia (red) serve as proxies for spinal cord segments. The location of dorsal root ganglia (DRG) and according sympathetic chain ganglia (SCHG) is indicated, but only faintly visible. Light sheet microscopy, 0.63× zoom factor. (b1) In a slice image, the individual dorsal root ganglia and according dorsal roots (white arrows) are visualized as they enter the spinal cord (SC), which defines the middle of the according spinal cord segment. Light sheet microscopy, 0.63× zoom factor. Note that lower sympathetic chain ganglia may serve as a proxy for spinal segments due to the increasing elongation of spinal roots before they enter the spinal cord [Color figure can be viewed at wileyonlinelibrary.com]

In order to allow imaging of preganglionic neurons in the SC, all animals received a laminectomy prior to staining procedures. We imaged specimens from their dorsal side with either light sheet or confocal microscopy (Figure 5a). PRV labeling was predominant in the ipsilateral IML, but we also noted consistent PRV labeling in the intercalated (IC) and central autonomic (CA) nuclei and some contralateral cells (Figure 5a). It should be noted that PRV tracing from peripheral organs to the SC also labels interneurons in addition to sympathetic preganglionic neurons, due to further propagation of the transsynaptic PRV infection (Schramm, Strack, Platt, & Loewy, 1993; Vera & Nadelhaft, 2000; Vizzard, Brisson, & de Groat, 2000). In line with that, and because tracer labeling was absent in contralateral SChG, we interpreted PRV labeled contralateral neurons as interneurons.

**FIGURE 6:**
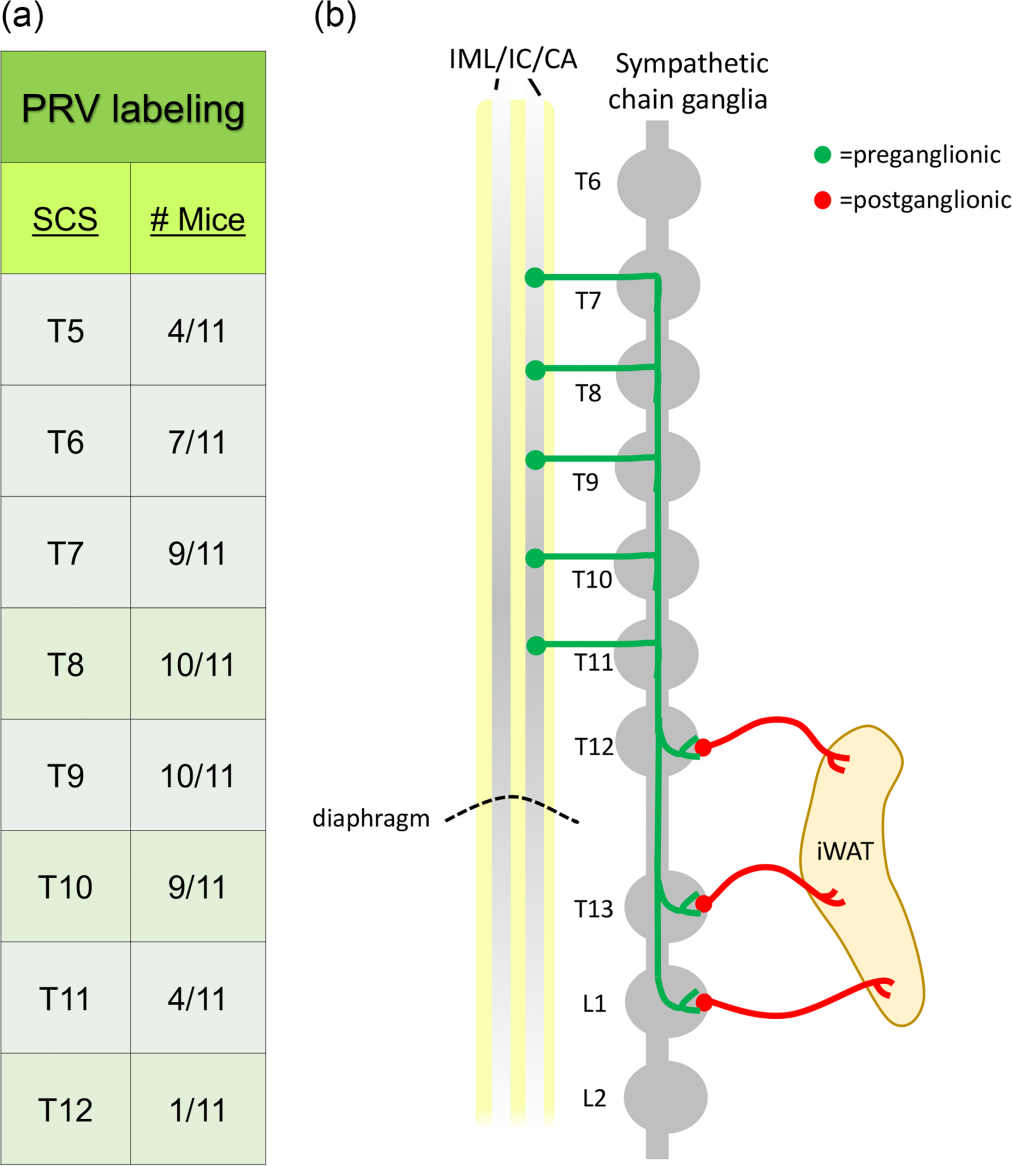
Preganglionic and postganglionic innervation of iWAT. (a) Diagrammatic representation of spinal cord segments (SCS) with PRV‐positive preganglionic neurons (*n* positive/*n* total number of mice) in IML, IC, and CA. Preganglionic iWAT innervation is observed from T5 to T12, but is most consistent across animals from T7 to T10. (b): Diagrammatic synopsis of preganglionic (green) and postganglionic (red) iWAT innervation. Note that the diagram only includes consistent pre‐ and postganglionic labeling sites. Also note the significant segmental caudal shift of postganglionic PRV labeling with respect to preganglionic innervation. CA, central autonomic; IC, intercalated; IML, intermediolateral nucleus of the spinal cord; iWAT, inguinal white adipose tissue; PRV, pseudorabies virus [Color figure can be viewed at wileyonlinelibrary.com]

The spinal root entry defines the middle of the according spinal segment. In three mice, we verified that thoracic SChG from T1 to T12 correlate well with the level of the DRG (Figure 5b) and the according dorsal root entry into the SC, which is shifted slightly rostral (Figure 5c). Thus, for data analysis of PRV labeling in iWAT related preganglionic neurons we used SChG as a proxy to determine their segmental level. Preganglionic labeling was restricted to the thoracic SC, with consistent labeling at T7–T10 in the majority of analyzed animals (82–91%), even though some cases included more rostral (T5) or caudal (T11) labeling (36%) (Figure 6a, Table 2d). Thus, iWAT‐related preganglionic neurons (T7–T10) show a significant segmental rostral shift with respect to postganglionic PRV‐labeled neurons (T13–L1) as highlighted schematically in Figure 6b.

### 3.5 Dissection and iDISCO clearing reveal two major access routes of sympathetic nerves to iWAT

**FIGURE 7:**
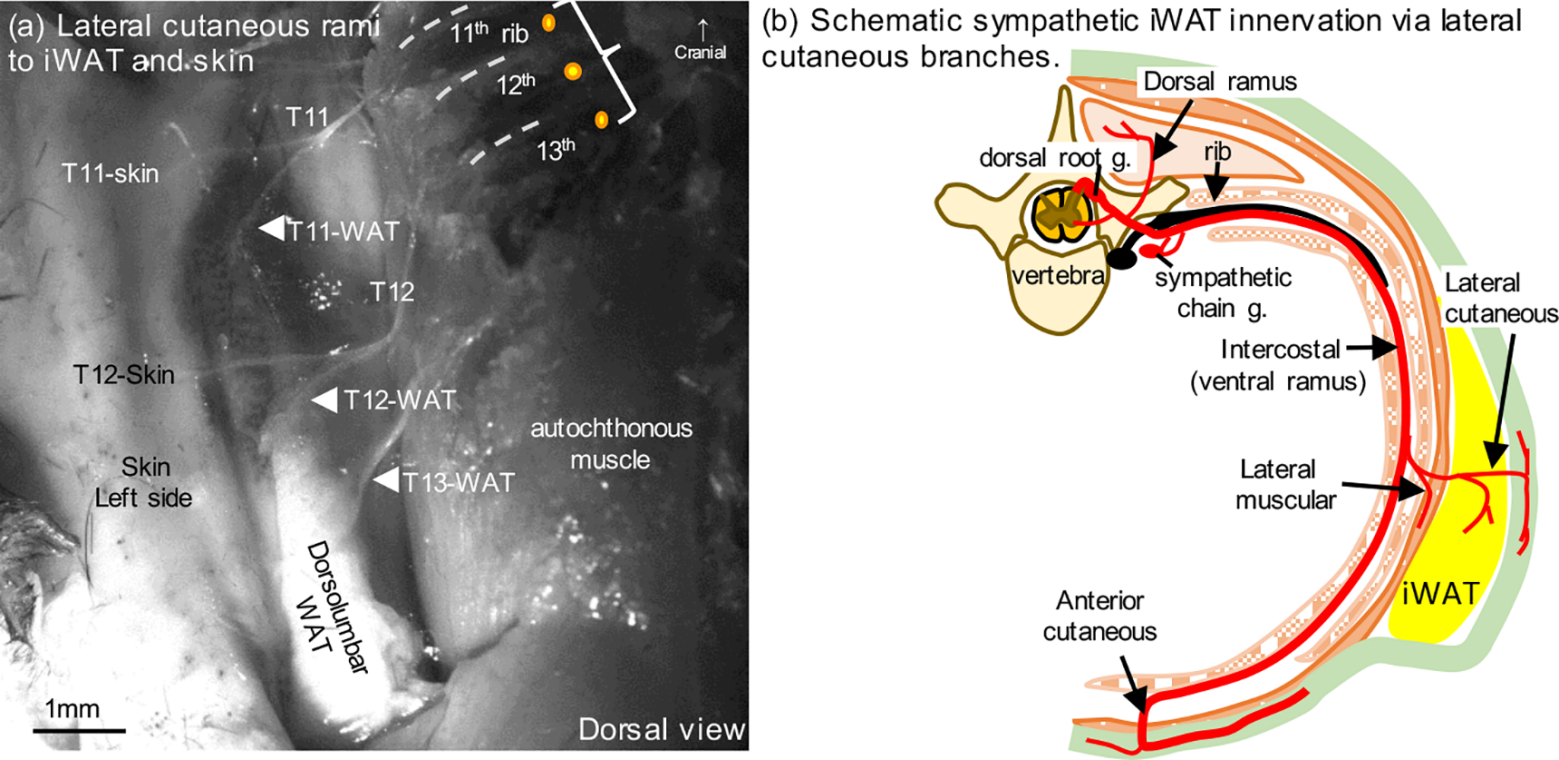
Sympathetic chain ganglia T11–T13 provide sympathetic innervation to dorsolumbar WAT via lateral cutaneous nerves. (a) Distribution of lateral cutaneous rami of intercostal nerves T11–T13 to dorsolateral iWAT (dl) as seen under the stereomicroscope (0.5× objective). Yellow dots indicate exit of dorsal cutaneous rami T11–T13 through the external oblique abdominal muscle (exobl) and fascia as landmarks for spinal nerve identification. (b) Scheme of lower thoracic spinal nerve branching emphasizing lateral cutaneous branches to iWAT. iWAT, inguinal white adipose tissue; WAT, white adipose tissue [Color figure can be viewed at wileyonlinelibrary.com]

**FIGURE 8:**
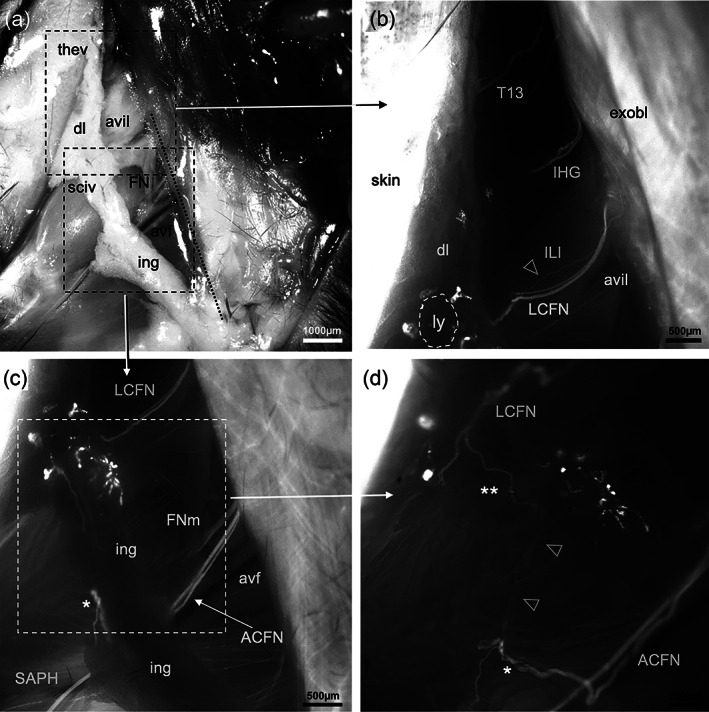
Peripheral nerves to right iWAT in a 3 week old female TH:tomato mouse in brightfield (a) and fluorescence (b–d) stereomicroscope, ventral view. (a) Brightfield overview (0.5× objective) of the right dorsolateral (dl) and inguinal (ing) portions of iWAT. The thoracoepigastric vein (thev) is visible at the cranial tip of the dl and the superficial ilium circumflex vein (sicv) is visible at the dl/ing iWAT junction. The femoral nerve (FN) appears upon its passage under the inguinal ligament (dotted) through the muscular lacuna. Medially, the femoral artery and vein (avf) are passing through the vascular lacuna. (b) The upper rectangle in (a) imaged at higher magnification under the fluorescent microscope (0.5× objective). Intercostal (subcostal) T13, iliohypogastric (IHG), the fine ilioinguinal (ILI, open arrowheads) and lateral cutaneous femoral (LCFN) nerves and the iliolumbar artery and vein (avil) travel to the dorsolateral portion. Dashed contour indicates lymph node (ly) at dl/ing junction; exobl denotes external oblique abdominal muscle and fascia. (c): Lower rectangle of (a) imaged at higher magnification under the fluorescent microscope (0.5× objective). The LCFN is seen approaching the dl/ing junction of iWAT. The main branches of the femoral nerve (ACFN, anterior cutaneous femoral nerve; FNm, motor branch to thigh extensor muscles; SAPH, saphenous nerve) are visible. The ACFN enters the inguinal portion and eventually exits to skin (cut, asterisk). (d) The rectangle in (c) imaged at higher magnification. Upon removing the overlying WAT, the LCFN divides. One branch (double asterisk) anastomoses with a fine branch of ACFN (open arrowhead) deep within the inguinal portion. Bright fluorescent artifacts at dl/ing junction typically occur after longer exposure on WAT surface. iWAT, inguinal white adipose tissue; TH, tyrosine hydroxylase; WAT, inguinal white adipose tissue

Next, we aimed to define the peripheral neural pathway, which can guide sympathetic postganglionic axons to the iWAT depot. Initially, we used bright field‐guided dissection and imaging to identify the sympathetic nerve inputs to dorsolumbar iWAT. We located the exit points of lateral cutaneous rami from intercostal nerves T11–T13 through the fascia and traced them to iWAT and skin (Figure 7a), as highlighted schematically in Figure 7b. The dorsolateral portion of iWAT is further innervated by the ilioinguinal (ILI) and the lateral cutaneous femoral nerves (LCFN, Figure 8a,b), which are both derived from the lumbar plexus.

The inguinal portion of the iWAT is entirely innervated by nerves that are derived from the lumbar plexus, which provides overall innervation to the ventral thigh. Figure 8c shows the inguinal portion of the iWAT in more detail. The major nerves visible in this view are the femoral nerve mainly derived from ventral rami of spinal nerves (L1–L3), from which the anterior cutaneous femoral nerve (ACFN) branches off and enters the inguinal division of the iWAT depot (Figure 8c). Further details of the innervation are visible upon removal of the overlying WAT, showing further branching of the LCFN and anastomoses with a branch of the ACFN (Figure 8d).

**FIGURE 9:**
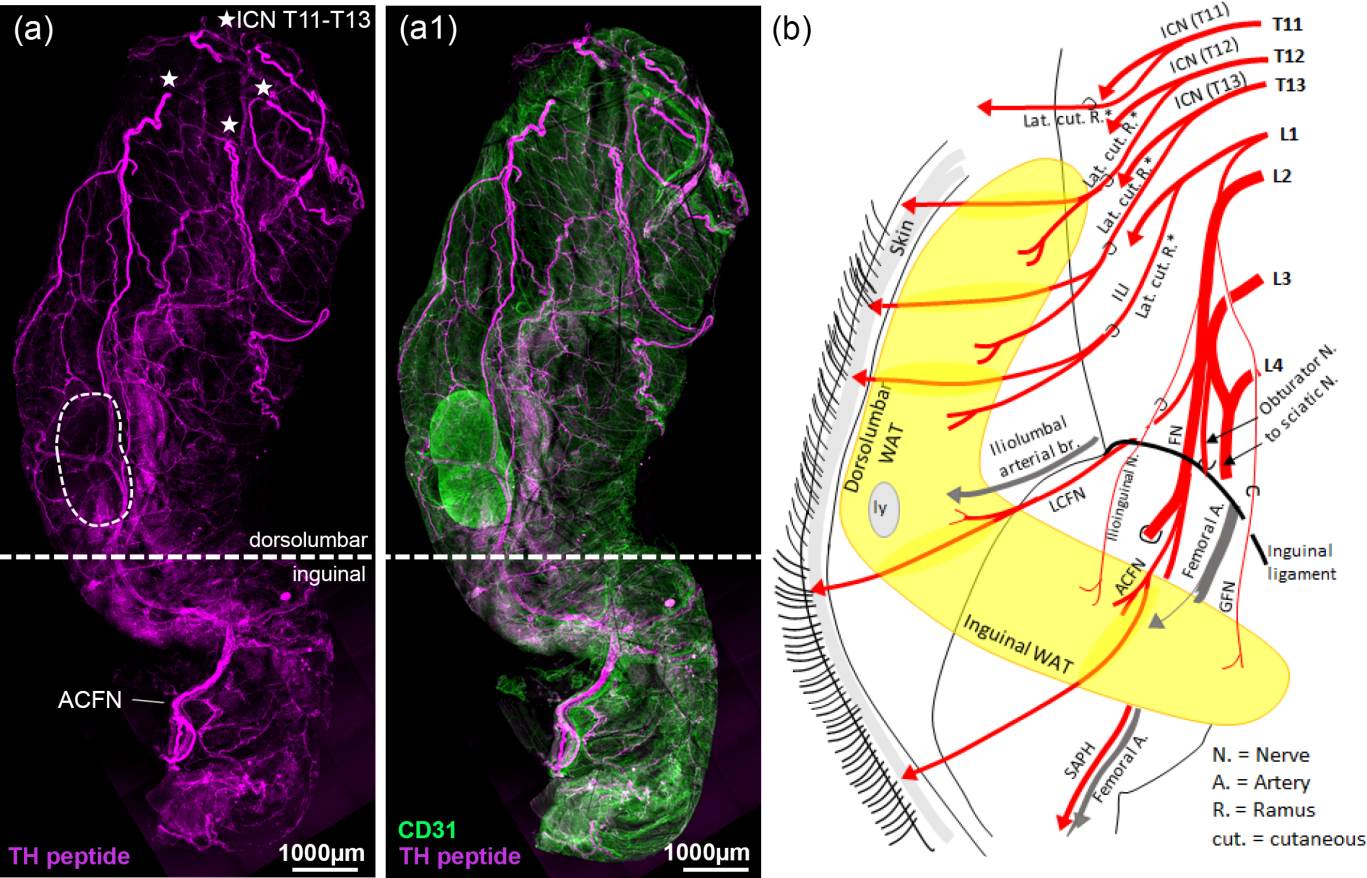
iDISCO tissue clearing shows main innervation branches to iWAT. (a) Confocal microscope images (5× objective) of an iWAT depot, stained and cleared with iDISCO for TH‐peptide (red = sympathetic innervation) and CD31 (green = vasculature and lymph node (ly)). The dorsolateral innervation shows several incoming branches (asterisk) that cannot be conclusively assigned to individual nerves, but should represent lateral cutaneous rami from T11 to T13. In the inguinal portion one prominent incoming branch can be consistently identified that we conclude is the anterior cutaneous femoral nerve (ACFN), which is also the most prominent nerve visible under the stereomicroscope. (b): Schematic drawing of the lumbar plexus that provides innervation to the thigh, including adipose tissue, vasculature and skin. GFN, genitofemoral nerve; ICN, intercostal nerve; iWAT, inguinal white adipose tissue; other abbreviations see legend to Figure 8

The complexity of the intercostal nerves and lumbar plexus nerves carrying sympathetic postganglionic fibers is not fully represented by stereomicroscopic images. Thus, we further dissected the entire iWAT depot and performed iDISCO staining for TH peptide and CD31 for better visualization of incoming major nerve fibers and vasculature (Figure 9a, a1). For orientation, we adopted the anatomical labeling and distinction of two iWAT portions that are roughly separated by a prominent lymph node in the WAT depot (Chi et al., 2018): the dorsolumbar and inguinal depot. We consistently observed one major, prominent nerve entering the inguinal portion of iWAT that is consistent with the anterior cutaneous femoral nerve (Figure 8c). In the dorsolumbar portion, we identified several incoming nerves (Figure 7a depicts three incoming nerves with asterisk). We found no consistency in the anatomical orientation of these nerves within dissected iWAT tissue, but they are consistent with the lateral cutaneous rami T11–T13 documented earlier (Figure 7a).

Furthermore, we assembled a schematic of the intercostal and lumbar plexus nerves as observed by stereomicroscope dissection in the mouse (Figure 7b). We found that all lateral cutaneous rami of intercostal nerves T11–T13 and cutaneous rami of the lumbar plexus provide innervation to the adipose tissue, skin, and vasculature. Thus, these innervation systems seem highly intertwined and should receive consideration specifically in denervation studies where innervation of vasculature, skin, and adipocytes will be likely similarly affected.

### 3.6 Varicose innervation density is sparse among white adipocytes, but dense among brown adipocytes within iWAT

**FIGURE 10:**
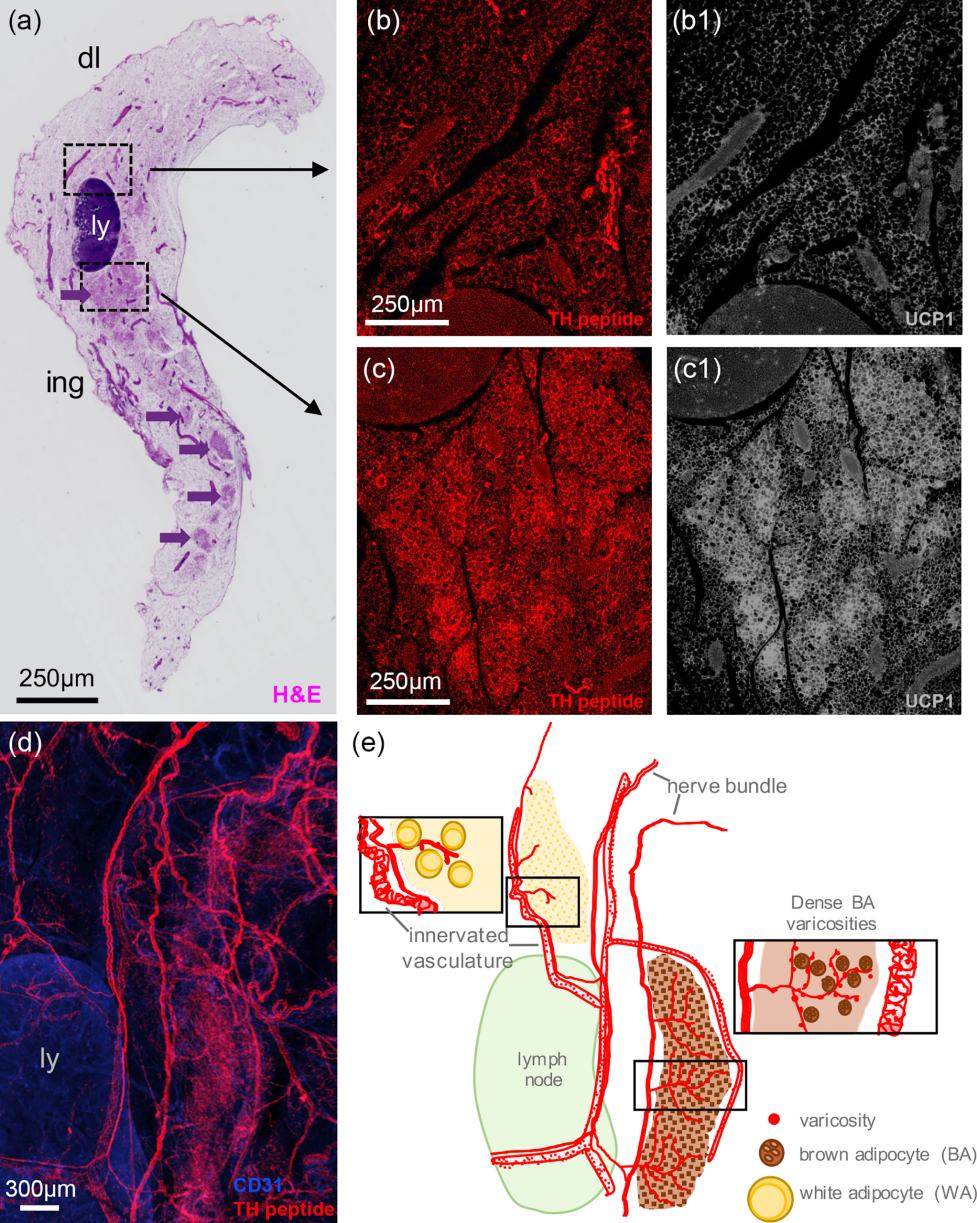
Varicose innervation density depends on white versus brown adipocyte innervation. (a) Hematoxilin and eosin staining (stains cytoplasmatic content purple) of a paraffin, longitudinal section (5 μm) of iWAT shows the prominent lymph node (ly) at the junction of dorsolumbar (dl) and inguinal (ing) portions of iWAT (confocal microscopy, 5× objective). Areas with mainly white adipocytes appear light purple (rectangle to (b)), due to the reduced cytoplasmatic content of white adipocytes. Areas with darker purple stain depict areas with increased number of brown adipocytes (beige islands, purple arrows and rectangle to (c)), which contain more coplasm than white adipocytes. The upper square depicts an area with mostly white adipocytes. Staining of an adjacent section with TH‐peptide (b) and the brown adipocyte marker Uncoupling protein 1 (UCP1) (b1) shows that low TH‐positive varicosities are found among UCP1‐negative white adipocytes. The lower square depicts an area with mostly brown adipocytes. Staining of an adjacent section with TH‐peptide (c) and UCP1 (c1) shows that high TH‐positive varicosities are found among UCP1‐positive brown adipocytes. (d) Confocal image (5× objective) of iWAT iDISCO cleared and stained for TH‐peptide and the vascular marker CD31. Apart from the major TH‐positive nerves, a more grainy TH labeling of axonal varicosities is seen. Co‐localization with CD31 shows dense varicosites associated with arterial vasculature. Beige islands with brown adipocytes can be identified by dense varicosities, while areas with mostly white adipocytes show low varicosity density. (e) The schematic drawing of the confocal image in (d) clarifies the observed pattern of axonal varicosites associated with arteries, brown and white adipocytes. iWAT, inguinal white adipose tissue; TH, tyrosine hydroxylase

Apart from the prominent main innervation branches depicted in Figure 7a, a grainier TH‐positive structure is visible. This structure seemed to represent areas with increased density of axonal varicosities, even though the resolution of light sheet or confocal images in cleared tissue samples was insufficient to fully resolve this. In a paraffin, longitudinal section through the iWAT the location of beige islands, that show increased accumulation of brown adipocytes (also known as beige or brite adipocytes), is visible (Figure 10a, purple arrows). Immunohistochemical staining of adjacent sections for TH‐peptide and uncoupling protein 1 (UCP1) demonstrates the stark difference in axonal varicosity density of UCP1 void areas (Figure 10b,b1) versus UCP1‐rich areas (Figure 10c,c1). Further details of an iDISCO cleared iWAT sample further depicts an area that shows neuronal branching with axonal varicosities into a beige island in close proximity to the lymph node (Figure 10d). For clarity, the image was reproduced as a schematic in Figure 10e.

## 4 DISCUSSION

Our study aimed to provide a comprehensive evaluation of preganglionic and postganglionic sympathetic inputs to iWAT in male and female mice, which allows the reconstruction of anatomical ganglia levels from their undisturbed location in whole body images. We could not find any obvious differences between male and female mice at any level of iWAT innervation. Our goal was also that our work would be useful for future studies that use virus‐driven neuromodulation in select adipose tissue depots.

Our PRV‐based study demonstrates SChG T12–L1 as major postganglionic input to iWAT, and is similar to data in other rodent models (Wiedmann et al., 2017; Youngstrom & Bartness, 1995). Recent reports have suggested that iWAT in mice may receive significant innervation by the celiac complex (Cao, Wang, & Zeng, 2018; Jiang et al., 2017), even though an innervation of iWAT from the celiac ganglia would be unorthodox as the celiac ganglia are rather indicated to provide sympathetic innervation for intra‐abdominal structures like liver, kidney, and gastrointestinal organs (Miolan & Niel, 1996). In our study, we rarely found labeling within celiac ganglia or the celiac‐superior mesenteric complex. For those rare cases we cannot exclude that some tracer may have diffused from subcutaneous depots through the vaginal process (in postnatal rodents typically open [Ramasamy et al., 2001]) as we previously suspected for claimed vagal innervation of iWAT (Berthoud et al., 2006). Specifically, large injection volumes and long incubation times may enhance the risk of diffusion through the inguinal canal, eventually labeling CG neurons that innervate intraperitoneal organs. Indeed, Jiang et al used a total tracer volume of 5 μl choleratoxin B with a 7‐day incubation time compared to a total PRV injection volume of 1–2 μl and 4 days incubation time in the present study. This further highlights the importance of our study to define reproducible labeling patterns for iWAT innervation, so that future approaches for viral targeting of the peripheral nervous system continues the same stringency as established for virus spread in the central nervous system (CNS) (Abbott, Machado, Geerling, & Saper, 2016; Chamberlin, Du, de Lacalle, & Saper, 1998; Gautron, Lazarus, Scott, Saper, & Elmquist, 2010; Hao et al., 2015). Another consideration for this unorthodox celiac ganglia labeling from iWAT would be via the genitofemoral nerve which we show in Figure 9b to innervate the medial iWAT tip and enters the abdominal cavity through the inguinal canal. Nevertheless, the genitofemoral nerve is a branch of the lumbar plexus and a neuronal connectivity with prevertebral ganglia has not been described so far. Also, while the iWAT may not receive direct innervation from the celiac ganglia, abdominal WAT depots, specifically those associated with the GI tract, liver and kidney are likely to receive innervation from the CG. Those are interesting aspects that will need to be addressed in future studies.

The findings presented here confirm postganglionic innervation from T13 and L1, which was found by others in rats and hamsters (Wiedmann et al., 2017; Youngstrom & Bartness, 1995). Another study in the hamster also found sympathetic inputs to iWAT from T12–L3, and additionally input from T1–T3 chain ganglia, with extensive overlap of sympathetic input to iBAT and iWAT (Nguyen et al., 2017). In contrast, the present study showed a more restricted input to iWAT (SChG T12–L1). None of our animals showed PRV labeling in rostral thoracic SChG (stellate/T1–T5), which we demonstrated recently as the main sympathetic input to iBAT (Francois et al., 2019; Münzberg et al., 2019). Importantly, our data suggest an anatomical separation of postganglionic input to iBAT versus iWAT. We speculate that the discrepancies of our study with Nguyen et al (Nguyen et al., 2017) are likely due to the extended PRV incubation time (6 days vs. 4 days in our study), as their aim was also to study overlapping patterns in CNS structures that require longer incubation time. Thus, while the ability of PRV for transsynaptic labeling is an advantage to identify circuits, it is important to recognize the possibility for transsynaptic labeling within preganglionic and postganglionic neurons with increasing incubation time and has been demonstrated for SC interneurons (Schramm et al., 1993; Vera & Nadelhaft, 2000; Vizzard et al., 2000).

We further show that the preganglionic input to iWAT (T7–T10) was also anatomically distinct from preganglionic input to iBAT (T2–T6) (Francois et al., 2019; Münzberg et al., 2019). We were unable to find other published work that anatomically defined the rostrocaudal extent of preganglionic input to iWAT or iBAT in the SC. Importantly, our data suggest that both preganglionic and postganglionic inputs to iBAT and iWAT are anatomically separated.

Our data provide the anatomical basis to suggest that CNS inputs could differentially regulate the sympathetic tone to iBAT versus iWAT. This is in line with a recent study indicating that hypothalamic *proopiomelanocortin* (Pomc) expressing neurons and *agouti‐related peptide* (Agrp) expressing neurons both regulate the sympathetic tone to iBAT, while sympathetic tone to iWAT was only regulated by Pomc neurons, but not Agrp neurons (Bell et al., 2018). This is also relevant, as several studies have now suggested that sympathetic activation of BAT might not be sufficient to induce BAT thermogenesis and might require additional sympathetic activation of WAT (Schreiber et al., 2017; Shin et al., 2017; Susulic et al., 1995). Future studies will need to address if known preganglionic inputs from the CNS may selectively regulate iBAT and iWAT sympathetic circuits. The clear anatomical separation of both preganglionic and postganglionic neurons innervating iWAT and iBAT, respectively, may facilitate future experimental studies on differential forebrain control of brown and WATs.

Sympathetic postganglionic axons travel via dorsal and ventral rami to their end organs, which combine sensory fibers from dorsal root ganglia and, via communicating rami, postganglionic fibers from SChG. Our anatomical dissections show that major incoming nerves to the dorsolumbar portion of iWAT are lateral cutaneous branches of intercostal nerves. Indeed, iWAT‐related PRV labeling of SChG is consistent with the innervation from intercostal nerves T12 to L1, which represent ventral rami of spinal nerves in that area.

Likewise, we found that the inguinal portion of iWAT is innervated via nerves derived from the lumbar plexus, which is also formed by ventral rami of spinal nerves, with a branch of the femoral nerve (L1–L3) and the lateral cutaneous nerve of the thigh (L2). iWAT‐related postganglionic neurons in chain ganglia L1–L3 send their axon through the communicating rami into these nerves. However, also lower thoracic chain ganglia, for example, T12 and T13 may connect to the lumbar plexus nerve, as a particular spinal nerve receives postganglionic axons from both, the next cranial and also the next caudal chain ganglia (Baron et al., 1988). As no PRV‐labeled postganglionic neurons were found in sympathetic ganglia caudal to L2, we speculate that this reflects PRV concentration into the dorsolumbar iWAT, which is consistent with our dorsal injection approach that might have unintentionally favored viral uptake into nerves to the dorsolumbar iWAT. Also, we cannot rule out that fat mass expansion with aging or obesity could further recruit innervation from more rostral or caudal SChG.

Interestingly, it has been noted by others that the dorsolumbar and inguinal portions show functional distinctions (Barreau et al., 2016): The inguinal portion is more prone to beiging and shows denser sympathetic innervation than the dorsolumbar portion. Thus, it is interesting to speculate if and how the anterior femoral nerve contributes to the increased beiging. Our data are the first to provide detailed maps for sympathetic nerves of iWAT. We further translate these data to 3D iDISCO imaging data in iWAT, so that a more targeted investigation and distinction of dorsolumbar versus inguinal innervation patterns is encouraged for future studies.

Importantly, varicose innervation density is strongly associated with adipose tissue structure. In iWAT, dense varicosities are mainly associated with arteries, while white adipocytes show sparse varicose innervation. In contrast, brown adipocytes clustered in beige islands, strongly correlate with increased varicose innervation pattern. Thus, dynamic interaction of neurotrophic repulsion and attraction, similar to autonomic innervation guidance during development (Glebova & Ginty, 2005; Thiede‐Stan & Schwab, 2015), are likely responsible for the robust difference in innervation patterns of white and brown adipocytes, respectively. However, the dynamic changes of varicose endings at the interface of white and brown adipocytes remain unstudied. Some studies used the dense network of sympathetic nerves to measure iWAT innervation density (Chi et al., 2018; Jiang et al., 2017), but this may not well represent the varicose innervation pattern at the interface of white and brown adipocytes.

Our data also clarify that nerves supplying the iWAT are continuous structures that transition through the adipose tissue further to the skin. Fine nerves branch off from these rami to reach deeper into the adipose tissue and provide a dense plexus of varicose axons especially to the smooth muscle media of small arteries and arterioles. Varicosities are sparse adjacent to white adipocytes, even though beige islands with brown adipocytes show a visibly denser varicosity pattern. It should be noted that surgical iWAT denervation, likely by cutting the lateral cutaneous rami and lumbar plexus incoming nerves (Vaughan, Zarebidaki, Ehlen, & Bartness, 2014), will remove innervation to iWAT, but also to the skin and vasculature. To selectively cut iWAT‐specific branches off the rami is likely not feasible, and therefore the confounding effects of skin and vasculature denervation should be considered in future surgical denervation studies. Alternatively, chemical denervation has been successfully used with comparable results to surgical denervation (Demas & Bartness, 2001), where the chemical toxin is directly injected into the adipose tissue depot. We predict that this method is more suitable to selectively denervate adipose tissue, while sparing innervation to further downstream structures like the skin that are jointly innervated by the same rami. We also suggest that future denervation studies (surgical or chemical) to include histological verification of the denervation extent with a focus on structures that share innervation via the same rami.

Furthermore, the precise anatomical identification of preganglionic and postganglionic innervation in the mouse should be a guide to verify correct viral labeling, for example, using virus‐driven expression of chemogenetic or optogenetic constructs, that are based on retrograde tracing methods. Furthermore, animals should be inspected for off‐target labeling in the CG, as they are inconsistent with iWAT innervation. Also, we currently have little understanding if all iWAT innervating nerves serve the same purpose, and past electric recordings of adipose tissue innervation lacked anatomical definition which nerves were recorded and if they included skin and vascular innervation or if more specific iWAT related recording is feasible (Bell et al., 2018). Our work enables future studies to provide anatomical detail about the nerves targeted. Another consideration is also the density of axonal varicosities as they are variable within the iWAT depot with dense varicosities in areas with high brown adipocytes (beige islands) and sparse varicosities adjacent to white adipocytes. We speculate that the density of varicosities could affect viral uptake and retrograde tracing efficiency. Indeed, in an earlier study we found that PRV incubation times after injection into the iBAT required less incubation time (Francois et al., 2019) than after injection into iWAT. Such dynamics will require more careful consideration in the peripheral nervous system when comparing mouse models with increased or decreased innervation (e.g., due to obesity or chronic cold exposure).

In summary, our data provide a comprehensive study to precisely define preganglionic and postganglionic sympathetic inputs to inguinal and dorsolumbar adipose iWAT tissue in male and female mice that are distinct from preganglionic and postganglionic inputs to BAT. These studies should serve as an anatomical guide to predict and verify proper viral infections for molecular genetic tracing of adipose tissue specific innervation, which is comparable to the exacting approach commonly used in CNS studies.

## CONFLICT OF INTEREST

The authors declare no potential conflict of interest.

## AUTHOR CONTRIBUTIONS

**Clara Huesing**: Data curation, formal analysis, investigation, visualization, writing—original draft preparation. **Emily Qualls‐Creekmore**: Data curation, investigation, writing—review and editing. **Nathan Lee**: Data curation, investigation. **Marie François**: Data curation, investigation. **Hayden Torres**: Data curation, formal analysis, investigation. **Rui Zhang**: Data curation, investigation. **David H Burk**: Funding acquisition, investigation, resources, writing—review and editing. **Sangho Yu**: Writing—review and editing, **Christopher D Morrison**: Writing—review and editing. **Hans‐Rudolf Berthoud**: Project administration, supervision, visualization, writing—review and editing. **Winfried Neuhuber**: Supervision, visualization, writing—review and editing. **Heike Münzberg**: Conceptualization, data curation, funding acquisition, investigation, project administration, resources, supervision, visualization, writing—original draft preparation.

### PEER REVIEW

The peer review history for this article is available at https://publons.com/publon/10.1002/cne.25031.

## Data Availability

The data that support the findings of this study are openly available on Blackfynn Discover at https://doi.org/10.26275/c4lt-jua2.
